# Dopamine Receptor Subtypes Differentially Regulate Autophagy

**DOI:** 10.3390/ijms19051540

**Published:** 2018-05-22

**Authors:** Dongmei Wang, Xinmiao Ji, Juanjuan Liu, Zhiyuan Li, Xin Zhang

**Affiliations:** 1 High Magnetic Field Laboratory, Key Laboratory of High Magnetic Field and Ion Beam Physical Biology, Hefei Institutes of Physical Science, Chinese Academy of Sciences, Hefei 230031, China; dongmeiwang@hmfl.ac.cn (D.W.); xinmiaoji@hmfl.ac.cn (X.J.); liujj@hmfl.ac.cn (J.L.); 2 Science Island Branch of Graduate School, University of Science and Technology of China, Hefei 230031, China; 3 Institute of Physical Science and Information Technology, Anhui University, Hefei 230601, China

**Keywords:** dopamine receptor, autophagy, AKT, mTOR, AMPK

## Abstract

Some dopamine receptor subtypes were reported to participate in autophagy regulation, but their exact functions and mechanisms are still unclear. Here we found that dopamine receptors D2 and D3 (D2-like family) are positive regulators of autophagy, while dopamine receptors D1 and D5 (D1-like family) are negative regulators. Furthermore, dopamine and ammonia, the two reported endogenous ligands of dopamine receptors, both can induce dopamine receptor internalization and degradation. In addition, we found that AKT (protein kinase B)-mTOR (mechanistic target of rapamycin) and AMPK (AMP-activated protein kinase) pathways are involved in DRD3 (dopamine receptor D3) regulated autophagy. Moreover, autophagy machinery perturbation inhibited DRD3 degradation and increased DRD3 oligomer. Therefore, our study investigated the functions and mechanisms of dopamine receptors in autophagy regulation, which not only provides insights into better understanding of some dopamine receptor-related neurodegeneration diseases, but also sheds light on their potential treatment in combination with autophagy or mTOR pathway modulations.

## 1. Introduction

Autophagy is an evolutionarily conserved process that degrades unwanted proteins, cytosol and organelles to maintain cellular and organism homeostasis [1]. Autophagy is executed by autophagy related proteins (ATGs) that are responsible for phagophore formation, nucleation, autophagosome elongation and closure. Among those, ATG8/LC3, the widely used autophagy marker, transforms from cytosolic LC3-I to membrane bound LC3-II when autophagy is induced [2]. Autophagy inhibitors such chloroquine (CQ) and bafilomycin A1 (Baf A1) are frequently used to evaluate the autophagic flux via LC3 turnover assay [3]. Besides ATGs, there are several proteins that can regulate autophagy. For example, AMPK and mTOR, which are essential players of cellular energy balance and organismal growth and homeostasis, could regulate autophagy in response to energy and nutrient availability [4,5]. AMPK consists of α, β, γ subunits and the α-T172 and β-S108 are the main phosphorylation sites for AMPK activity [6,7]. mTOR signaling pathway involves the upstream PI3K-AKT and downstream p70 S6K and 4E-BP1 substrates [8,9], and its dysregulation is associated with numerous human diseases [10,11,12,13].

Dopamine receptors (DR), including D1-5 (also called DRD1-5), are originally identified to be the receptors for dopamine, an endogenous neurotransmitter that controls a variety of brain functions, including emotion, cognition and movement [14,15,16]. As a GPCR (G protein-coupled receptor) member, DR is classified into D1-like (including DRD1 and DRD5) and D2-like (including DRD2, DRD3 and DRD4) families according to their coupled G proteins, Gαs or Gαi [17,18]. Many neurogenic diseases such as Parkinson and Alzheimer’s disease were associated with DR dysfunction and relevant agonists or antagonists are used to target DR for therapy or modified to generate effective probes for live imaging [19,20,21,22,23]. Since Zhang et al. reported that compounds with affinity for DRs could modulate autophagy in a screen [24], functions of different DRs agonists and antagonists in autophagy have been examined in cells and animals [25,26,27,28,29,30,31]. However, the DR subtype functions in autophagy by themselves were not studied directly.

We previously found that ammonia is an endogenous ligand for DRD3. In addition to its role of inhibiting autophagic flux by modulating intra-vesicular pH, ammonia could also induce autophagy through DRD3 and mTOR [32]. In this paper, we systematically studied the roles of different DR subtypes in autophagy and further investigated the intertwined regulation between DRD3 and autophagy, which seems to be related to AKT-mTOR and AMPK pathways.

## 2. Results

### 2.1. Dopamine Receptors D2 and D3 (D2-Like Family) Are Positive Regulators of Autophagy

**Figure 1:**
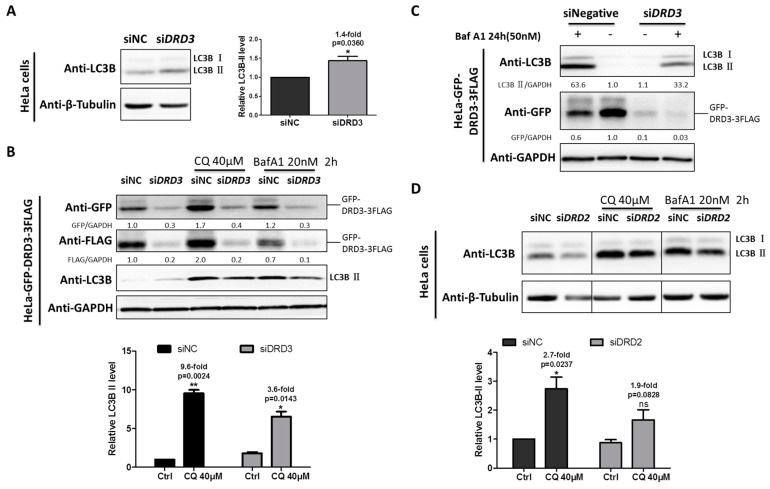
DRD3 and DRD2 knockdown inhibit autophagic flux. (**A**) Dopamine receptor D3 (DRD3) RNAi in HeLa cells was used to test the relative level of LC3B-II. (**B**) DRD3 RNAi was combined with autophagy inhibitors Chloroquine (CQ) (40 μM) or Baf A1 (20 nM) for 2 h to detect the autophagic flux in HeLa cells stably expressing GFP-DRD3-3FLAG. (**C**) DRD3 RNAi was combined with high concentration of Baf A1 (50 nM) for 24 h to examine the autophagic flux in HeLa cells stably expressing GFP-DRD3-3FLAG. (**D**) DRD2 RNAi in HeLa cells was combined with autophagy inhibitors CQ (40 μM) or Baf A1 (20 nM) for 2 h to detect the autophagic flux. Experiments were repeated at least three times and representative Western blots are shown. Densitometric analysis was performed and quantification results were labeled below the corresponding blots. * *p* < 0.05, ** *p* < 0.01.

We previously dissected the mechanism of ammonia-induced autophagy through dopamine receptors D3 (DRD3) and mTOR [32]. To investigate the exact role of DRD3 itself in autophagy, DRD3 knockdown and autophagy inhibitors were combined to examine autophagic flux level changes. When DRD3 was knocked down by DRD3 RNAi, the relative LC3B-II, the autophagosome-bound LC3, was slightly increased (Figure 1A). However, the increased LC3B-II may be the result of increased autophagy induction or decreased autophagic degradation [2]. It has been well accepted that the autophagic flux could be more accurately shown by differences in the relative level of LC3-II between samples in the presence and absence of autophagy inhibitors [2]. In order to examine the autophagic flux in DRD3 knockdown cells, LC3 turnover assay using autophagy inhibitors CQ or Baf A1 was performed [3]. Our results show that the autophagic flux was obviously decreased in the DRD3 RNAi group compared with the negative control group (Figure 1B). Furthermore, the differences were more significant when higher concentration of Baf A1 and prolonged treatment time were used (Figure 1C). These evidences show that DRD3 is a positive regulator of autophagy.

Given that both DRD2 and DRD3 belong to D2-like family of DR, we next examined the role of DRD2 in autophagy regulation. Similarly, LC3 turnover assay also was performed between the negative control and DRD2 RNAi groups as DRD3 RNAi, which indicated that DRD2 knockdown also inhibited autophagic flux shown by the relative differences of LC3B-II (Figure 1D), which implies that DRD2 is a positive regulator of autophagy as well.

### 2.2. Dopamine Receptors D1 and D5 (D1-Like Family) Are Negative Regulators of Autophagy

**Figure 2:**
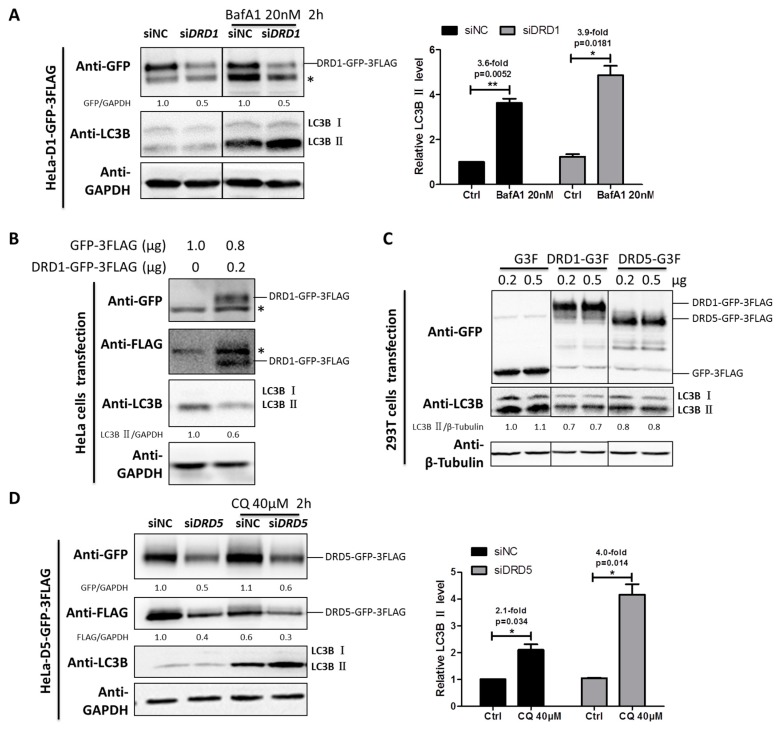
DRD1 and DRD5 knockdown promote autophagic flux. (**A**) DRD1 RNAi was combined with autophagy inhibitor Baf A1 (20 nM) for 2 h to detect the autophagic flux in HeLa cells stably expressing DRD1-GFP-3FLAG. (**B**) Total of 1 μg of MSCV-DRD1-GFP-3FLAG and MSCV-GFP-3FLAG plasmids were transfected into HeLa cells using lipofectamine 2000 for 48 h. (**C**) 0.2 or 0.5 μg MSCV-DRD1/DRD5-GFP-3FLAG or MSCV-GFP-3FLAG plasmid was transfected into 293T cells using lipofectamine 2000 for 48 h. (**D**) DRD5 RNAi was combined with autophagy inhibitors CQ (40 μM) for 2 h to detect the autophagic flux in HeLa cells stably expressing DRD5-GFP-3FLAG. The asterisk (*) indicates the nonspecific band. Experiments were repeated at least three times and representative Western blots are shown. Densitometric analysis was performed and quantification results were labeled below the corresponding blots or in separate panels. * *p* < 0.05, ** *p* < 0.01.

DRD1 and DRD5 belong to D1-like family, and they are functionally different from the D2-like family members. To investigate the roles of DRD1 and DRD5 in autophagy regulation, HeLa cells stably expressing DRD1 and DRD5 were established using MSCV infection (Figure S1). Furthermore, in order to examine the effect of DRD1 knockdown on autophagic flux, Baf A1 combined with DRD1 RNAi induced higher LC3-II level than the negative control, indicating increased autophagic flux after DRD1 knockdown (Figure 2A). Next we overexpressed DRD1 in HeLa cells and found the DRD1 expression levels were associated with LC3B-II levels (Figure 2B). Moreover, GFP-3FLAG tagged DRD1 was also transiently expressed in 293T cells (Figure 2C), and it was obvious that DRD1 expression decreased LC3B-II in 293T cells as well (Figure 2C), which was consistent with the results in HeLa cells (Figure 2B). Therefore, DRD1 knockdown and overexpression experiments in HeLa and 293T cells all show that DRD1 is a negative regulator of autophagy.

As for the role of DRD5 in autophagy, we also combined overexpression and knockdown experiments. GFP-3FLAG tagged DRD5 was transiently transfected into 293T cells and the LC3-II level was obviously decreased compared to vector control (Figure 2C). We also performed LC3 turnover assay in DRD5 knockdown cells using autophagy inhibitor CQ. It was interesting that DRD5 knockdown could increase the LC3-II level in CQ treated cells, indicating increased autophagic flux. Therefore, DRD5 overexpression and knockdown experiments both show that DRD5 is a negative regulator of autophagy, which is similar to the other D1-like member, DRD1 (Figure 2C,D).

### 2.3. Both Dopamine and Ammonia Induce Dopamine Receptor Degradation

**Figure 3:**
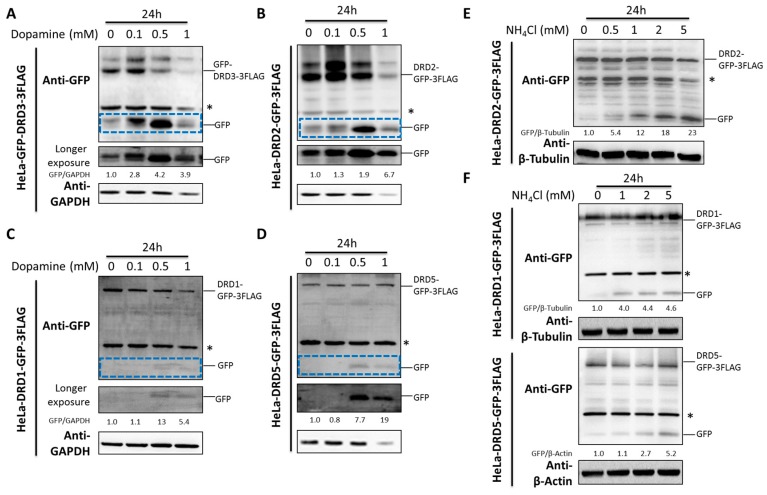
Dopamine and ammonia induce the degradation of D1-like and D2-like dopamine receptors differentially. (**A**–**D**) HeLa cells stably expressing GFP-3FLAG tagged DRD1, 2, 3 and 5 were treated with different concentrations of dopamine for 24 h. (**E**,**F**) HeLa cells stably expressing GFP-3FLAG tagged DRD1, 2 and 5 were treated with different concentrations of ammonia for 24 h. The asterisk (*) indicates the nonspecific band. Experiments were repeated at least three times and representative Western blots are shown. Blue dashed frames show the GFP fragment with shorter exposure. Densitometric analysis was performed and quantification results were labeled below the corresponding blots.

Dopamine is the well-known endogenous ligand for dopamine receptors. Due to the fact that some ligands could induce the degradation of their receptors [33,34], we therefore studied the effects of dopamine on dopamine receptor degradation. Notably, dopamine induced the D2-like family DRD2 and DRD3 degradation and the GFP fragment accumulation from GFP tagged DRD2 or DRD3 (Figure 3A,B). However, the D1-like family DRD1 and DRD5 were much less affected compared with the D2-like family (Figure 3C,D).

Ammonia, a recently discovered endogenous ligand for DRD3, was shown to induce significant DRD3 degradation and GFP fragment accumulation from GFP tagged DRD3 [32]. Here we examined its effects on the degradation of other dopamine receptors. It is interesting that ammonia induced significant GFP fragment accumulation from GFP tagged DRD2 (Figure 3E), which is similar to DRD3. However, its effect on the D1-like family DRD1 and DRD5 were not as significant as the D2-like family (Figure 3F), which was consistent with the effects of dopamine. Hence, both dopamine and ammonia could induce significant degradation of D2-like family DRs but only moderately affect the D1-like family. In another word, the D2-like family DRs seem to be more sensitive to their endogenous ligand-induced degradation than the D1-like family DRs.

### 2.4. Dopamine and Monoamines Are DRD3 Ligands and Induce DRD3 Internalization and LC3B Increase

**Figure 4:**
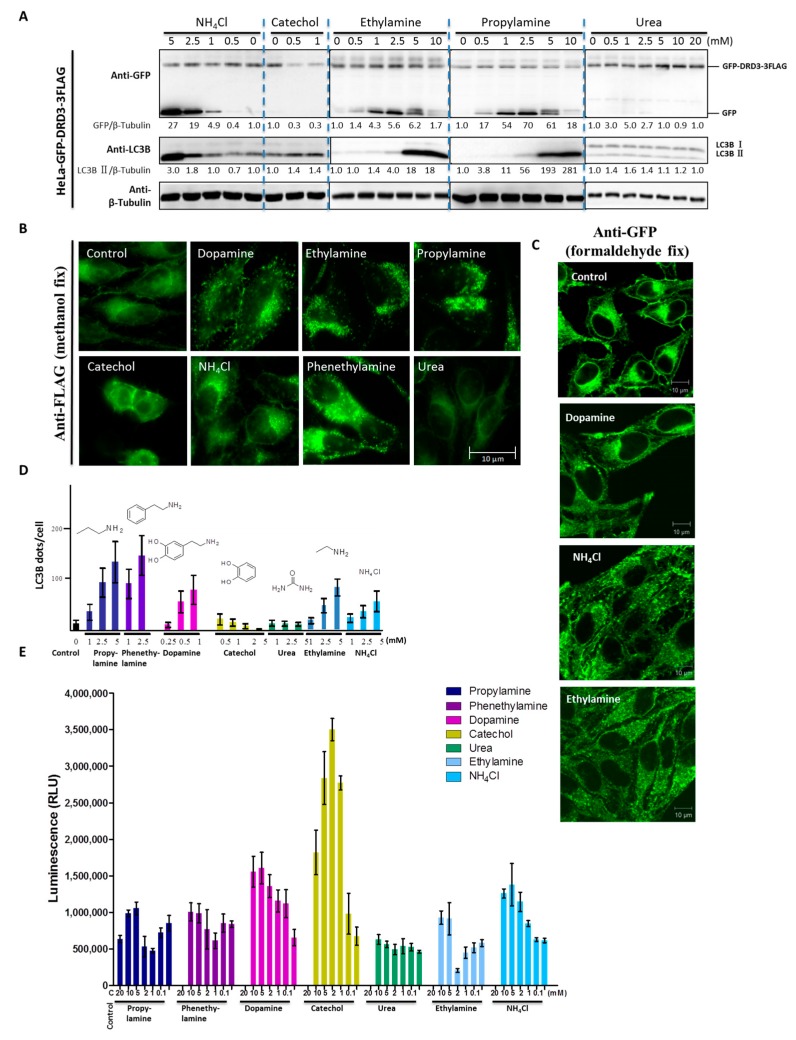
The free amino group is responsible for dopamine and monoamine-induced autophagy and DRD3 internalization. (**A**) Representative Western blots show GFP fragment and autophagy levels upon addition of different chemicals. The blue dashed lines are helpful to distinguish different lanes in each group. HeLa cells stably expressing GFP-DRD3-3FLAG were treated with different concentrations of catechol, NH_4_Cl, ethylamine, propylamine or urea for 24 h. Densitometric analysis was performed and quantification results were labeled below the corresponding blots. (**B**) GFP-DRD3-3FLAG localizations in cells treated with different chemicals. Immunofluorescence used anti-FLAG antibody to analyze the localization of GFP-DRD3-3FLAG. HeLa cells stably expressing GFP-DRD3-3FLAG were treated with dopamine, catechol, NH_4_Cl, ethylamine, propylamine, phenethylamine or urea for 24 h. Cells were fixed with methanol, blocked by AbDil-Tx (containing 0.1% Triton X-100) and then subjected to anti-FLAG antibody staining. Experiments were repeated at least three times and representative results are shown. Scale bar, 10 μm. (**C**) GFP-DRD3-3FLAG localizations in cells treated with dopamine and monoamines. Cells treated as Figure 4B were fixed with 3.7% formaldehyde, blocked by AbDil-Tx (containing 0.1% Triton X-100) and then subjected to anti-GFP antibody staining. Experiments were repeated at least three times and representative results are shown. Scale bar, 10 μm. (**D**) LC3B puncta in cells treated with different chemicals. Quantification results of the LC3B puncta in HeLa cells stably expressing GFP-DRD3-3FLAG treated with different concentrations of ammonia, dopamine, urea, catechol and some monoamines. (**E**) cAMP responses in cells treated with different chemicals. cAMP-Glo experiment was used to measure cAMP level in CHO cells that stably express GFP-DRD3-FLAG upon adding NH_4_Cl, dopamine, catechol, urea, ethylamine, propylamine or phenethylamine. Data show mean ± SD from three independent experiments.

Previously, we found that DRD3 is a receptor for ammonia-induced autophagy [32]. Then we pursued structure-activity analysis of DRD3 ligands, where we quantified the induction of autophagy by candidate DRD3 ligands. DRD3’s endogenous ligand dopamine contains amine and catechol functional groups, suggesting that DRD3 might bind and sense these moieties. We first tested the effect of dopamine, catechol and ammonium chloride on cells and found that although dopamine and ammonium chloride increased cleaved GFP fragment, catechol did not (Figure 3A and Figure 4A). This indicates that the ligand-receptor recognition may act through the amine or ammonia functionality rather than the hydroxyl. Next, we examined several amine derivatives to find out whether other primary amines can also cause the same effect. Urea was used as a negative control because it is a carbamide, which does not have a free amino group. We found that ethylamine and propylamine both increased the cleaved GFP fragment while urea did not (Figure 4A). To compare their ability to induce GFP-DRD3-3FLAG internalization and modulate autophagy, we examined the localization of GFP-DRD3-3FLAG (Figure 4B). However, since the methanol fix procedure can not differentiate internal antigens from external ones because the methanol permeabilizes the cell membrane, we fixed the cells using formaldehyde and also permeabilized them using 0.1% Triton X-100 to perform immuno-staining. In this way, the membrane part can be better preserved than methanol fix and internalized antigens could be detected simultaneously. As shown in Figure 4C, the signals from cell surface were reduced in dopamine and monoamines treated groups compared with control group, which suggested the internalization of surface GFP-DRD3-3FLAG (Figure 4C). It also indicates similar internalization induced by dopamine and monoamines as in methanol fix (Figure 4B,C). Also, we analyzed LC3B puncta (Figure 4D) in cells that were treated as in Figure 4B. Our results show that propylamine and phenethylamine have the strongest phenotype, while catechol and urea do not increase GFP-DRD3-3FLAG internalization (Figure 4B) or LC3B puncta in cells (Figure 4D). To measure the downstream G protein mediated traditional GPCR signaling pathway, we used cAMP assays to measure the effects of these potential ligands. Consistent with the autophagy results, urea does not affect the cAMP level in CHO-GFP-DRD3-FLAG cells (Figure 4E). However, cAMP assay results of the other ligands do not completely correlate with autophagy induction. For example, catechol also induces cAMP changes similar to dopamine and the ethylamine, while propylamine and phenethylamine-induced cAMP change is much weaker than NH_4_Cl (Figure 4E). These indicate that dopamine and monoamines are all DRD3 ligands and they could induce DRD3 internalization and degradation, as well as LC3B increase, which confirms the role of DRD3 in autophagy. 

### 2.5. AKT-mTOR and AMPK Are Involved in DRD3-Regulated Autophagy

**Figure 5:**
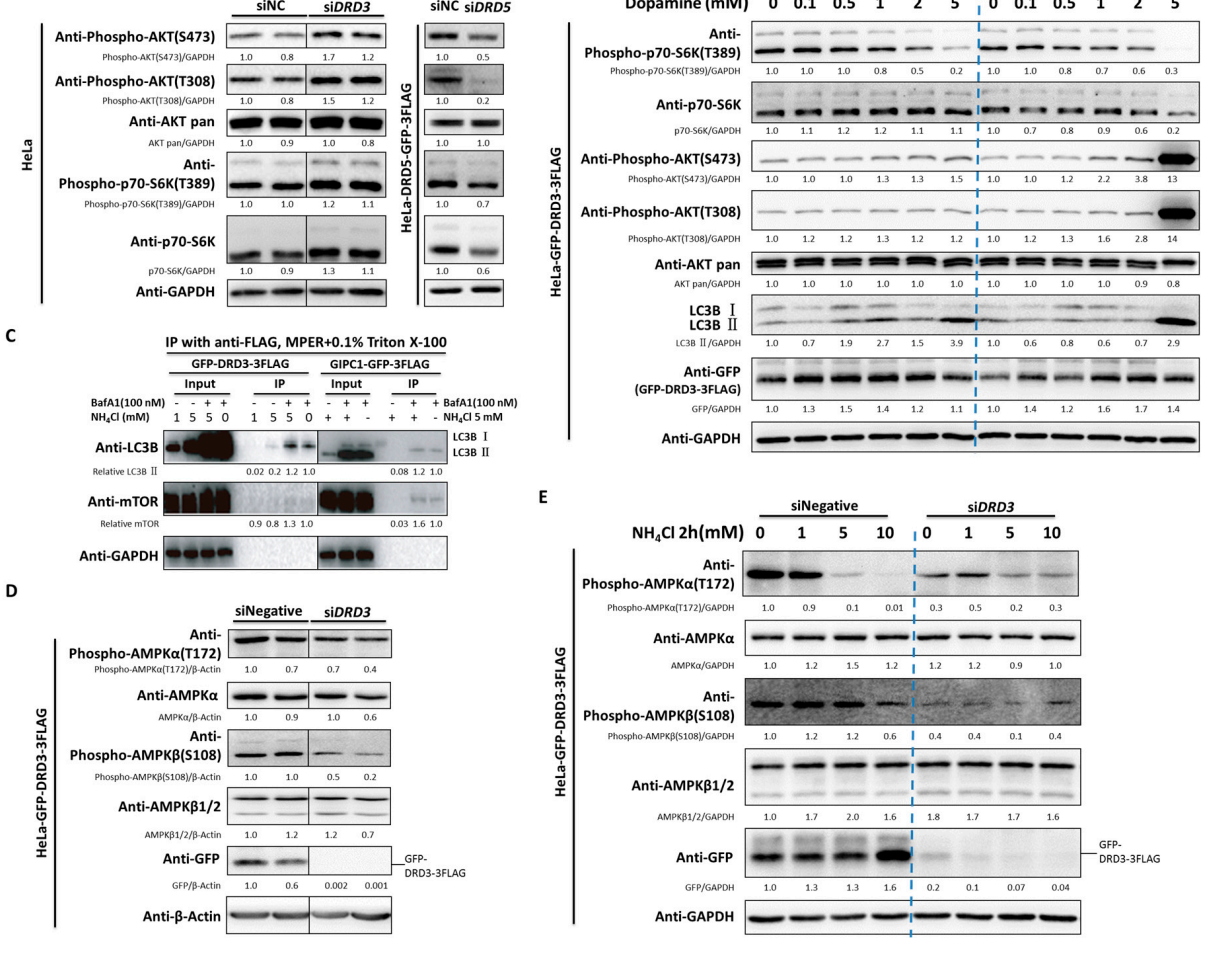
AKT (protein kinase B)-mTOR (mechanistic target of rapamycin) and AMPK (AMP-activated protein kinase) pathways are involved in DRD3-regulated autophagy. (**A**) DRD3 or DRD5 RNAi in HeLa wild type or HeLa cells stably expressing DRD5-GFP-3FLAG. (**B**) HeLa cells stably expressing GFP-DRD3-3FLAG were treated with increasing concentrations of dopamine for 1 h or 8 h. (**C**) Co-Immunoprecipitation using anti-FLAG in HeLa cells stably expressing GFP-DRD3-3FLAG or GIPC1-GFP-3FLAG treated with Baf A1 and/or NH_4_Cl, in the presence of additional 0.1% Triton X-100 in IP and washing buffers. (**D**) DRD3 RNAi in HeLa cells stably expressing GFP-DRD3-3FLAG decreases AMPK activity shown by AMPKα-T172 and β-S108. (**E**) DRD3 RNAi in HeLa cells stably expressing GFP-DRD3-3FLAG partially antagonizes the effect of ammonia-induced AMPKα-T172 and β-S108 inhibition. The blue dashed lines are used to distinguish different parts of the results for better visualization. Experiments were repeated at least three times and representative Western blots are shown. Densitometric analysis was performed and quantification results were labeled below the corresponding blots.

To find out the downstream signaling pathways for dopamine receptor regulated autophagy, we chose DRD3 and DRD5 as representative D1-/D2-like family receptors for further investigation. Although dopamine agonist such as quinelorane activates PI3K-AKT-mTOR pathway [35,36], little is known for the roles of DR themselves in AKT-mTOR pathway. It was interesting that DRD3 knockdown increased AKT phosphorylation at both Ser-473 and Thr-308 while DRD5 knockdown showed opposite effects (Figure 5A). Furthermore, DRD3 knockdown increased the mTOR substrate phospho-p70-S6K (T389) level while DRD5 knockdown showed opposite effect as well (Figure 5A). These results indicate that the D1 and D2-like family dopamine receptors both can modulate the AKT-mTOR signaling pathway, but in an opposite way.

To further dissect the underlying mechanism for the autophagy regulation function of dopamine receptors, we chose DRD3 for further studies. Although dopamine was reported to regulate AKT-mTOR signaling in human SH-SY5Y neuroblastoma cells [37], the exact role of dopamine on DRD3 is unclear due to the fact that other DRs are also highly expressed in the neuroblastoma cells. Therefore, for their low expression of DRs, HeLa cells were selected for dopamine effects on downstream signaling. HeLa cells stably expressing GFP-DRD3-3FLAG were treated with dopamine or ammonia, the two reported DRD3 ligands, for different time points. Even as short as for 1 h treatment of dopamine could obviously inhibit the mTOR substrate phospho-p70-S6K (T389) phosphorylation. At 8 h, dopamine also induced AKT activation, which is likely due to the negative feedback loop of mTOR signaling excessive inactivation (Figure 5B). Consistently, ammonia also increased AKT phosphorylation (Figure S2) and decreased mTOR substrate phospho-p70-S6K (T389) level [32]. These results confirmed the involvement of AKT-mTOR pathway in dopamine receptor-regulated autophagy.

To further study the relationship between mTOR and dopamine receptors, we established a HeLa cell line stably expressing GFP-3FLAG tagged GIPC1 (GAIP interacting protein, C terminus), the downstream scaffold protein for DRD2 and DRD3. Considering that the basal levels of autophagosome protein LC3B is usually low in untreated cells, we used Baf A1 and ammonia to enrich autophagosomes. Using co-immunoprecipitation (co-IP) experiments, we added extra 0.1% Triton X-100 to the MPER buffer that already contained mild detergent to reduce nonspecific binding. It is obvious that LC3B and mTOR could be pulled out by both GFP-DRD3-FLAG and GIPC1-GFP-3FLAG (Figure 5C). The interaction between mTOR and DRD3 seem to be much weaker than mTOR and GIPC1, which implies that DRD3 may rely on GIPC1 to regulate mTOR.

In addition, we found that AMPK was also affected by DRD3. Specifically, AMPK activity was inhibited by DRD3 knockdown, which is shown by decreased phosphorylation level of AMPK α-T172 and β-S108 (Figure 5D). Interestingly, ammonia-induced AMPK signaling inhibition (Figure 5E) was partially antagonized by DRD3 knockdown, which avoided ammonia-induced excessive AMPK inhibition in DRD3 knockdown cells (Figure 5E, lane 4 and 8). Therefore, our results show that DRD3 knockdown could increase AKT-mTOR activity and decrease AMPK activity. Given that autophagy was regulated by the balance between mTOR and AMPK activity and AKT as the upstream kinase for mTOR [4,5,10], the AKT-mTOR and AMPK pathways might both contribute to the autophagy regulation by DRD3.

### 2.6. Perturbation of Autophagy Machinery Induced DRD3 Degradation Inhibition and Oligomer Increase

**Figure 6:**
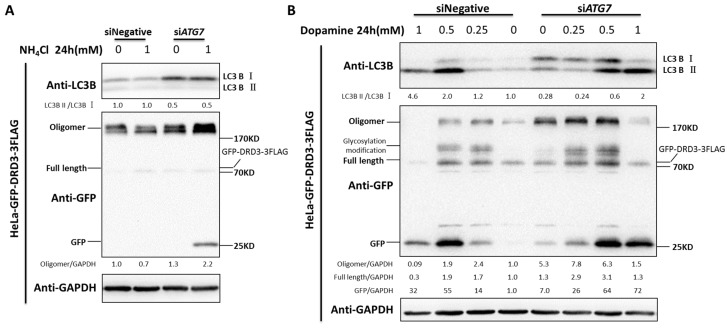
Autophagy inhibition decreases dopamine receptor degradation and increases oligomer formation. (**A**) ATG7 RNAi in HeLa cells stably expressing GFP-DRD3-3FLAG were treated with 1 mM ammonia for 24 h. (**B**) ATG7 RNAi in HeLa cells stably expressing GFP-DRD3-3FLAG were treated with different concentrations of dopamine for 24 h. Experiments were repeated at least three times and representative Western blots are shown. Densitometric analysis was performed and quantification results were labeled below the corresponding blots.

The GFP fragment is an intermediate degradation byproduct from GFP-DRD3 because GFP can be degraded in the lysosomes but not the intermediate autophagosomes [38]. At lower concentrations of ammonia, GFP fragment could not be easily detected due to lower GFP-DRD3 degradation rate and robust lysosomal degradation capacity. To further examine the role of autophagy in DRD3 degradation, the autophagy machinery was perturbed to test the DRD3 protein level changes. Knockdown of Beclin-1 or ATG7, two core autophagy components, significantly increased the GFP fragment in lower concentration of ammonia (1 mM, which is not sufficient to induce GFP-DRD3 degradation in control condition), which indicated the role of autophagy in DRD3 degradation (Supplementary Materials Figure S3A,B and Figure 6A). These results indicate that autophagy perturbation could sensitize GFP fragment accumulation, which may be due to the compromised autolysosomal degradation for intermediate GFP induced by lower concentration of ammonia.

In the meantime, we noticed that the full length of GFP-DRD3 protein level did not increase when autophagy machinery was perturbed (Figure 6A, Figure S3A,B). Given that DRD3 might form oligomers [39], we next examined the oligomer level before and after autophagy machinery perturbance. In fact, the DRD3 oligomer significantly increased after ATG7 knockdown, indicating the conditioned accumulation of DRD3 oligomer by autophagy perturbation (Figure 6A). Therefore, the oligomer-form of DRD3 should also be considered to quantify the total protein amount of DRD3. In addition, since dopamine could induce DRD3 degradation, we further examined the role of autophagy in dopamine-induced DRD3 degradation. The LC3B-I to LC3B-II conversion was also inhibited after ATG7 knockdown, indicating compromised autophagy flux (Figure 6A,B). In the meantime, after 1 mM of dopamine treatment, almost all GFP-DRD3-3FLAG proteins, including the full-length monomer form as well as the oligomer form, were degraded (Figure 6B). However, in ATG7 knockdown cells, there are still some GFP-DRD3-3FLAG monomer and oligomer left, which indicates that the ammonia-induced DRD3 degradation was inhibited by ATG7 knockdown. It was also interesting that ATG7 knockdown could alleviated the cytotoxicity of higher concentrations of dopamine [40], which is likely due to autophagic cell death.

## 3. Discussion

The different roles of D1-like and D2-like DR subtypes in autophagy regulation may be due to their differentially associated G protein and downstream scaffold proteins. D1-like DR subtypes DRD1 and DRD5 are coupled to Gαs G protein while D2-like DR subtypes DRD2 and DRD3 are coupled to Gαi G protein [17]. Interference of the Gαi with PTX (Pertussis toxin), locking Gαi in the GDP-bound inactive state, could induce autophagy [41]. In addition, D2-like DRD2 and DRD3 are associated with the scaffold protein GIPC and it has been evidenced that GIPC induced autophagy in pancreatic cancer cells [42,43,44]. We also attempted to clone DRD4 but did not succeed, which is probably due to the high GC content in DRD4 sequence. Moreover, as DRD4 belongs to D2-like family and its gene polymorphism [45] encodes different isoforms, here the complex role of DRD4 variants in autophagy was not discussed. In sum, the differential roles of D1-like and D2-like DRs in autophagy might be due to the different downstream signaling partners.

Some studies show that dopamine receptor agonists or antagonists are involved in autophagy regulation. For example, DRD4 antagonists such as L-741, 742 and PNU 96415E disrupted the autophagy-lysosomal pathway [30]; DRD5 agonists induced autophagy and autophagic cell death [29]; DRD2 antagonists such as raclopride and sertindole induced autophagy [26,31]. Although dopamine receptors agonists or antagonists participated in autophagy regulation, little was known about the exact roles of dopamine receptors themselves in autophagy. Hence, based on our previous report, our study here further confirms that DRs participate in autophagy regulation. 

Most GPCRs are internalized by endocytic sorting and degraded by the general lysosome pathway [46,47]. Moreover, brain cannabinoid 1 receptor has been shown to be degraded by autophagy [48]. However, whether DRs are degraded through autophagy pathway is still unknown. Our findings here provide evidences for the autophagic degradation of DRs, which will further strengthen the link between autophagy and GPCRs degradation.

The main finding of our study concerns that expression of DRD2 and DRD3, by itself, without interaction with ligands, induces autophagy, and that the opposite situation occurs in the case of DRD1 and DRD5. Considering the role of autophagy in neurodegenerative diseases [49,50,51], constitutive expressing DRs in some neurons might be responsible for the formation of misfolded proteins and neuro-degeneration. However, since there are many factors affecting autophagy, other proteins and environmental factors should also be considered for autophagy contribution in some neurodegenerative diseases, such as Parkinson’s disease and Huntington’s disease when using autophagy as a therapeutic strategy.

There are some evidences showing that DR could form oligomers, such D1-D2, D1-D3 and D2-adenosine A2A receptor [39,52,53,54,55,56]. Here we found that DRD3 preferentially existed as oligomers when autophagy was compromised, which may be a potential indicator for autophagy inhibition in DRD3 associated diseases. However, whether the accumulated DRD3 oligomers have specific function or just aggregate due to degradation inhibition is unknown. In addition, whether and how DRs form oligomers to regulate autophagy also needs to be investigated.

DRs were found as the receptors for dopamine and many neuro-degeneration diseases are associated with their dysfunction. In this paper, we systematically studied the roles of D1-like and D2-like family receptors in autophagy regulation. Our results show that D1-like family receptors DRD1 and DRD5 negatively regulate autophagy, while D2-like family receptors DRD2 and DRD3 positively regulate autophagy. DRD3 generally functions through the downstream cAMP associated signaling cascade to control intracellular events [57]. Here we found that the AKT-mTOR and AMPK pathways might participate in DRD3 regulated autophagy, which will provide some clues for the connection between DR and the intracellular signaling hub. Our findings not only revealed the role of DRD3 in autophagy but also connected DRD3 signaling with the cellular energy and nutrient sensor, mTOR and AMPK, which will broaden the scope of DRD3 study and guide combined therapeutics for DR associated diseases in the future.

## 4. Materials and Methods

### 4.1. Cell Culture and Stable Cell Lines Establishment

HeLa and 293T cells were cultured in DMEM (Corning Cellgro, 15-017-CVR, Manassas, VA, USA) supplemented with 10% Fetal Bovine Serum (CLARK Bioscience, Richmond, VA, USA), 2 mM GlutaMAX (Gibco, Carlsbad, CA, USA), 100 units/mL of Penicillin-100 µg/mL of Streptomycin (HyClone, Logan, UT, USA). HeLa-DRD1-GFP-3FLAG, HeLa-DRD2-GFP-3FLAG, HeLa-GFP-DRD3-3FLAG, HeLa-DRD5-GFP-3FLAG, HeLa-GIPC1-GFP-3FLAG cells were maintained in DMEM complete medium with 1 μg/mL puromycin (Selleck, Washington, DC, USA). HeLa-GFP-DRD3-3FLAG cells were established as previously described [32,58]. In addition, HeLa-DRD1/DRD2/DRD5/GIPC1-GFP-3FLAG cells were established similarly. The cDNA for DRD1/DRD2/DRD5/GIPC1 were amplified from HeLa cells and cloned into MSCV vector with GFP-3FLAG in their C-terminus.

### 4.2. Transient Transfection

HeLa or 293T cells were plated at 30% confluence in 12-well-plate 24 h before experiment. Then cells were transfected for the plasmids of MSCV-GFP-3FLAG, MSCV-DRD1/DRD5-GFP-3FLAG using lipofectamine 2000 (Invitrogen, Waltham, MA, USA) and cultured for another 48 h for Western blots.

### 4.3. RNAi for Dopamine Receptors, ATG7 and Beclin-1

The RNAi assay was conducted as described before [32]. Briefly, for one well in 12-well-plate, HeLa wild type or the dopamine receptors overexpression cell lines were trypsinized, plated and transfected with 6 μL Hiperfect (Qiagen, Dusseldorf , Germany) and 2.4 μL siRNAs (20 μM) in 100 μL opti-MEM (Gibco, Carlsbad, CA, USA) according to the manufacture’s protocol. After 72 h incubation, cells were lysed for Western blots. The sequences of siRNA oligos targeted to human mRNA were as below (5′-3′): Negative or NC: UUCUCCGAACGUGUCACGUTT; *DRD1*: GGACCUUGUCUGUACUCAUTT; *DRD2*: GAAGAAUGGGCAUGCCAAA; *DRD3*: GUACAGCCAGCAUCCUUAA; *DRD5*: GCAGUUCGCUCUAUACCAGTT; *ATG7*: CAACAUCCCUGGUUACAAG; *Beclin-1*: UAAGAUGGGUCUGAAAUUU.

### 4.4. Western Blots and Co-Immunoprecipitation

Cells were lysed on ice by the M-PER (Thermo Scientific, Waltham, MA, USA) supplemented with protease and phosphatase inhibitors cocktail (Roche, Basel, Switzerland) for 30 min. The whole cell lysate was denatured in the final 1× SDS loading buffer at 95 °C for 5 min. Then the denatured samples were subjected to the SDS-PAGE and subsequent Western blots such as PVDF membrane (Merck, Whitehouse Station, NJ, USA) transfer, primary and HRP-conjugated secondary antibodies incubation. For co-immunoprecipitation, cells were cultured in 10-cm-dish to 95–100% confluence and lysed the same as above. The anti-FLAG M2 monoclonal antibody was incubated with Dynabeads protein G (Invitrogen, Waltham, MA, USA) at room temperature for 45 min on a rotator. The whole cell lysate were centrifuged at 10,000× *g* for 1 min to get rid of cell debris and the supernatant was mixed with antibody-conjugated Dynabeads protein G at 4 °C for 45 min on a rotator. After washing with ice-cold PBST (PBS with 0.04% Tween-20) three times every 5 min at 4 °C (using the magnet to separate the Dynabeads mixture and the supernatant), the Dynabeads were supplemented with lysis buffer and SDS sample buffer followed by boiling at 95 °C for 5 min. And the supernatant was used as input control. The immunoprecipitates and input were subjected to subsequent SDS-PAGE and Western blots. The chemiluminesence results were obtained using Tanon Fine-do X6 (Shanghai, China) catalyzed by Thermo Scientific (Waltham, MA, USA) or Millipore ECL (Billerica, NJ, USA). Results shown in figures are all representative.

### 4.5. Immunofluorescence

HeLa cells expressing GFP-DRD3-3FLAG were grown on coverslips and treated with ammonia, dopamine, urea, catechol and some monoamines for 24 h. Cells were washed once with PBS and fixed by −20 °C methanol for 5 min or fixed with 3.7% formaldehyde at room temperature for 15 min and then blocked by AbDil-Tx (TBS-Tx supplemented with 0.1% Triton X-100, 2% BSA and 0.05% sodium azide) at room temperature for 30 min, followed by primary antibodies (FLAG, GFP or LC3B) incubation at 4 °C overnight. The secondary fluorescently conjugated antibodies were incubated at room temperature for 1 h and washed by TBS-Tx (TBS added with 0.1% Triton x-100) and mounted by anti-fade prolong Gold with DAPI (4′,6-Diamidino-2-Phenylindole, Dihydrochloride) (Invitrogen, Waltham, MA, USA). Images were taken using a Leica DMI4000B fluorescent microscope (Leica Camera, Wetzlar, Germany) or Zeiss LSM 710 confocal microscope (Carl Zeiss AG, Oberkochen, Germany). Images shown in figures are all representative results from multiple independent experiments.

### 4.6. Reagents

The autophagy antibody sampler kit, the antibodies for phospho-S6K (T389/412), S6K, AKT pan, phospho-AKT (Thr-308), phospho-AKT (Ser-473), mTOR, AMPK Antibody sampler kit, the HRP-linked anti-rabbit and anti-mouse IgG antibody were all from Cell signaling technology. The anti-GFP (sc-9996) antibodies were acquired from Santa Cruz. The anti-GAPDH, anti-β-Tubulin and anti-β-Actin antibodies were from Beijing TransGen Biotech (Beijing, China). Dynabeads Protein G was from NOVEX. The secondary antibodies and anti-fade prolong Gold with DAPI were from Molecular Probes. The anti-FLAG M2 monoclonal antibody (F3165), dopamine, chloroquine, NH_4_Cl were from Sigma. Ethylamine was from J&K Chemical (Shanghai, China), catechol from Energy Chemical (Shanghai, China), propylamine and phenethylamine from Tokyo Chemical Industry. GlutaMAX supplement was from Gibco. Puromycin dehydrochloride was from Selleck. Bafilomycin A1 was from Cayman. The siRNAs were ordered from GenePharma (Shanghai, China).

### 4.7. cAMP-Glo Assay

The intracellular cAMP level was monitored by the cAMP-Glo assay (Promega) based on the reciprocal relationship between the cAMP concentration and the bioluminescence value. The decreased luminescence reading reflects higher cAMP level in cells. Briefly, 5000 cells (CHO and CHO-GFP-DRD3-FLAG cells) were plated in white 384-well plate (Corning, Manassas, VA, USA, 3570) 24 h prior to the assay. Cells were washed once with PBS and then were pre-treated with 20 μL compounds of interest in PBS for 25 min before treated with 7.5 μL compounds in the presence of 1 mM IBMX, 200 μM Ro 20-1724 and 10 μM forskolin for 15 min at room temperature. The subsequent steps were performed as the manufacture’s protocol indicated. The data were acquired with the Multimode Plate Reader (EnVision, PerkinElmer, Waltham, MA, USA) and analyzed by GraphPad Prism 5 (GraphPad Software, La Jolla, CA, USA).

### 4.8. Statistical Analysis

ImageJ software (NIH, Bethesda, Maryland, USA) was used for densitometric analysis of Western blots to quantify the relative protein levels. GraphPad Prism 5 was used for Student’s *t*-test. *p* values < 0.05 were considered as statistically significant.

## Supplementary Materials

Supplementary Materials can be found at http://www.mdpi.com/1422-0067/19/5/1540/s1.

## Abbreviations

4E-BP1	Translation initiation factor 4E-binding protein 1
AKT	Protein kinase B
AMPK	AMP-activated protein kinase
ATG7	Autophagy related protein 7
LC3	Microtubule-associated protein 1 light chain 3
mTOR	Mechanistic/mammalian target of rapamycin
p70-S6K	Ribosomal protein S6 kinase beta-1
