# Supplementary material for: Dopamine Receptor Subtypes Differentially Regulate Autophagy
Source: Int J Mol Sci. 2018 May 22;19(5):1540. doi: 10.3390/ijms19051540 (PMC5983733; doi:10.3390/ijms19051540)
Supplement: Supplementary file 1 [file ijms-19-01540-s001.pdf]

Supplementary Materials

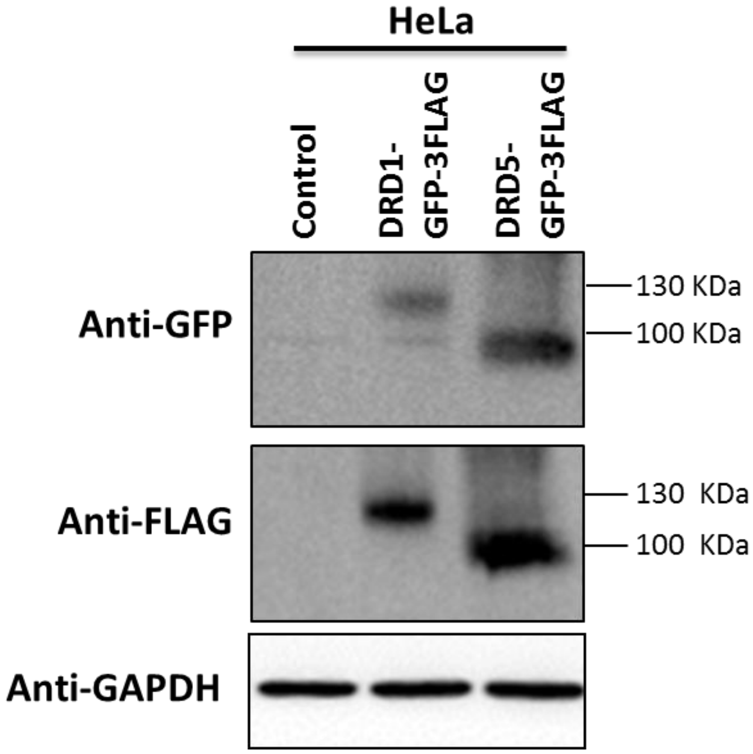

Figure S1. DRD1/DRD5-GFP-3Flag stably expressing HeLa cells were established with MSCV infection and verified by Western blot. Both anti-GFP and anti-Flag antibodies were used to detect the full length of DRD1/DRD5-GFP-3Flag. Representative Western blots are shown.

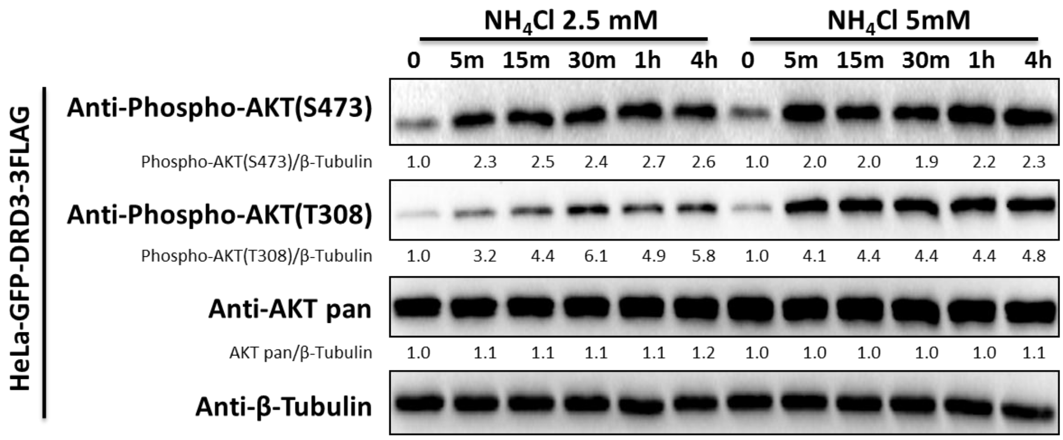

Figure S2. Ammonia increased AKT phosphorylation at Ser-473 and Thr-308. 2.5 and 5 mM ammonia treatment for different time was shown to increase ATK activity in GFP-DRD3-3Flag stably expressing HeLa cells. Representative Western blots are shown. Densitometric analysis was performed and quantification results were labeled below the corresponding blots.

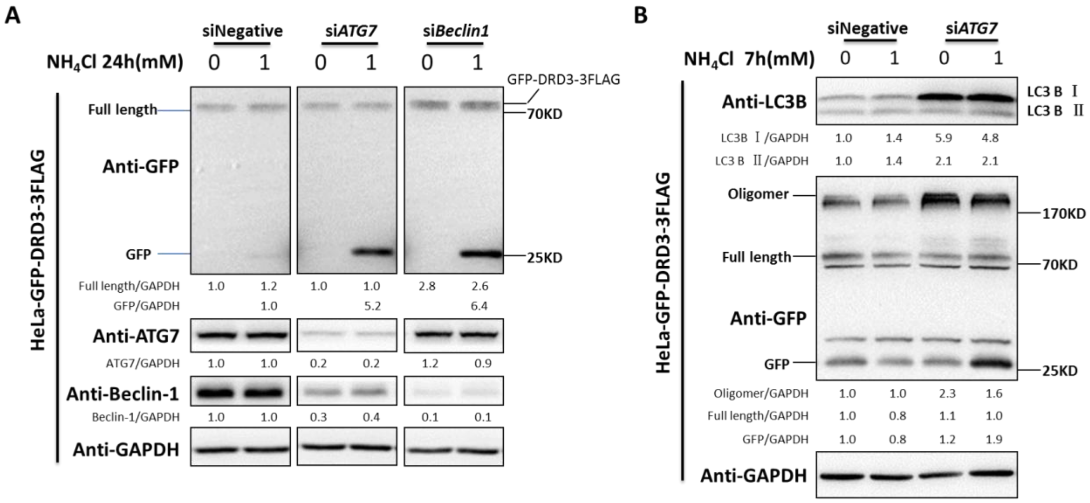

Figure S3. Autophagy machinery perturbance sensitizes the GFP fragment accumulation from GFP-DRD3 but does not affect GFP-DRD3-3Flag full length protein level. (**A-B**) ATG7 and/or Beclin-1 knockdown was performed in GFP-DRD3-3Flag stably expressing HeLa cells. Then the cells were treated with 1 mM ammonia for 24 h or 7 h and lysed for Western blot analysis. Representative Western blots are shown. Densitometric analysis was performed and quantification results were labeled below the corresponding blots.
